# Identification of groups with poor cost-effectiveness of peginterferon plus ribavirin for naïve hepatitis C patients with a real-world cohort and database

**DOI:** 10.1097/MD.0000000000006984

**Published:** 2017-06-02

**Authors:** Pei-Chien Tsai, Ta-Wei Liu, Yi-Shan Tsai, Yu-Min Ko, Kuan-Yu Chen, Ching-Chih Lin, Ching-I Huang, Po-Cheng Liang, Yi-Hung Lin, Ming-Yen Hsieh, Nai-Jen Hou, Chung-Feng Huang, Ming-Lun Yeh, Zu-Yau Lin, Shinn-Cherng Chen, Chia-Yen Dai, Wan-Long Chuang, Jee-Fu Huang, Ming-Lung Yu

**Affiliations:** a Hepatobiliary Division, Department of Internal Medicine, and Hepatitis Center, Kaohsiung Medical University Hospital; b Department of Internal Medicine, Kaohsiung Municipal Hsiao-Kang Hospital; c Faculty of Internal Medicine, College of Medicine, Kaohsiung Medical University; d Institute of Biomedical Sciences, National Sun Yat-Sen University, Kaohsiung, Taiwan.

**Keywords:** chronic hepatitis c, cost-effectiveness analysis, pegylated interferon, ribavirin, sustained virological response, treatment-naïve

## Abstract

**Background:**

For decades, peginterferon and ribavirin (PegIFN/RBV) have been the standard-of-care for chronic hepatitis C virus (CHC) infection. However, the actual cost-effectiveness of this therapy remains unclear. We purposed to explore the real-world cost effectiveness for subgroups of treatment-naïve CHC patients with PegIFN/RBV therapy in a large real-world cohort using a whole population database.

**Methods:**

A total of 1809 treatment-naïve chronic hepatitis C virus (HCV) patients (829 HCV genotype 1 [G1] and 980 HCV G2) treated with PegIFN/RBV therapies were linked to the National Health Insurance Research Database, covering the entire population of Taiwan from 1998 to 2013 to collect the total medical-care expenses of outpatient (antiviral agents, nonantiviral agents, laboratory, and consultation costs) and inpatient (medication, logistic, laboratory, and intervention costs) visits. The costs per treatment and the cost per sustained virological response (SVR) achieved were calculated.

**Results:**

The average medical-care cost was USD $4823 (±$2984) per treatment and $6105 (±$3778) per SVR achieved. With SVR rates of 68.6% and 87.8%, the cost/SVR was significantly higher in G1 than those in G2 patients, respectively ($8285 vs $4663, *P* < .001). Treatment-naïve G1 patients of old ages, those with advanced fibrosis, high viral loads, or interleukin-28B unfavorable genotypes, or those without a rapid virological response (RVR: undetectable HCV RNA at week 4), or those with complete early virological response (cEVR: undetectable HCV RNA at week 12). Treatment-naïve G2 patients with high viral loads or without RVR or cEVR incurred significantly higher costs per SVR than their counterparts. The cost/SVR was extremely high among patients without RVR and in patients without cEVR.

**Conclusion:**

We investigated the real-world cost effectiveness data for different subgroups of treatment-naïve HCV patients with PegIFN/RBV therapies, which could provide useful, informative evidence for making decisions regarding future therapeutic strategies comprising costly direct-acting antivirals.

## 1 Introduction

There are approximately 185 million people worldwide infected with hepatitis C virus (HCV),^[1,2]^ if left untreated, 10% to 15% of these individuals will develop cirrhosis,^[3]^ leading to life-threatening comorbidities such as liver decompensation and liver carcinoma with annual incidence rates of 2% to 6% and 1% to 5%,^[4–6]^ respectively. The age- and sex-adjusted prevalence of antibodies to HCV was estimated to be about 3.3% in Taiwanese,^[7]^ where several HCV hyperendemic areas exist with anti-HCV prevalence rates up to 30% to 60%.^[8,9]^

Pegylated interferon (PegIFN) plus ribavirin (RBV) double therapies have been the standard-of-care in the last 2 decades. It achieves higher sustained virological response (SVR) rates in Taiwan when compared to Western countries,^[10,11]^ because of a much higher percentage of the favorable interleukin-28B (IL28B) in East Asian patients.^[12,13]^ With genotype- and response-guided therapies, the SVR rate could achieve 75% for HCV genotype 1 (G1) and 85% to 90% for HCV G2 patients using PegIFN/RBV for 16 to 48 weeks.^[14,15]^ Therefore, the National Health Insurance Administration in Taiwan began to reimburse for PegIFN/RBV treatment for chronic HCV patients in 2003. However, adverse events resulting from PegIFN/RBV treatment continued to be a major issue requiring close monitoring, dose reductions, and even early discontinuation in the treatment of chronic hepatitis C^[16]^ and led to a huge gap between high clinical efficacy (80%) and little community effectiveness (13%) in Taiwan.^[7]^ Nevertheless, due to a relatively favorable conformation of HCV genotypes and IL28B SNPs,^[17,18]^ carefully chosen patient groups according to genotype,^[19]^ fibrosis stage,^[20]^ and IL28B allele^[21]^ boosted efficacy, ameliorated adverse events, and reduced medical expenses.

With the creation of IFN-free, direct-acting antiviral agents (DAAs) came the potential to avoid the adverse effects of IFN and RBV and offered promising efficacy over 90% to 100%.^[22–24]^ However, the new DAAs are expensive, necessitating a thorough cost-effectiveness analysis to compare treatments with different new DAAs and traditional PegIFN/RBV for reference in resource-constrained areas.^[25]^

The Taiwan National Health Insurance covered about 99.7% of the entire population since 1995, and it also provided a comprehensive database for cost-effectiveness analyses in the real world. In our pilot study of treatment-naïve outpatient cost data, we showed that the cost per SVR achieved was significantly higher in G1 patients than in G2 patients.^[26]^ Therefore, we want to conduct a large cohort, real-world cost effectiveness analysis with both of outpatient- and inpatient-information by linking a clinical cohort to the NHI research database (NHIRD) to investigate factors associated with the cost-effectiveness of PegIFN/RBV among treatment-naïve chronic hepatitis C (CHC) patients.

## 2 Materials and methods

### 2.1 Study population

**Figure 1:**
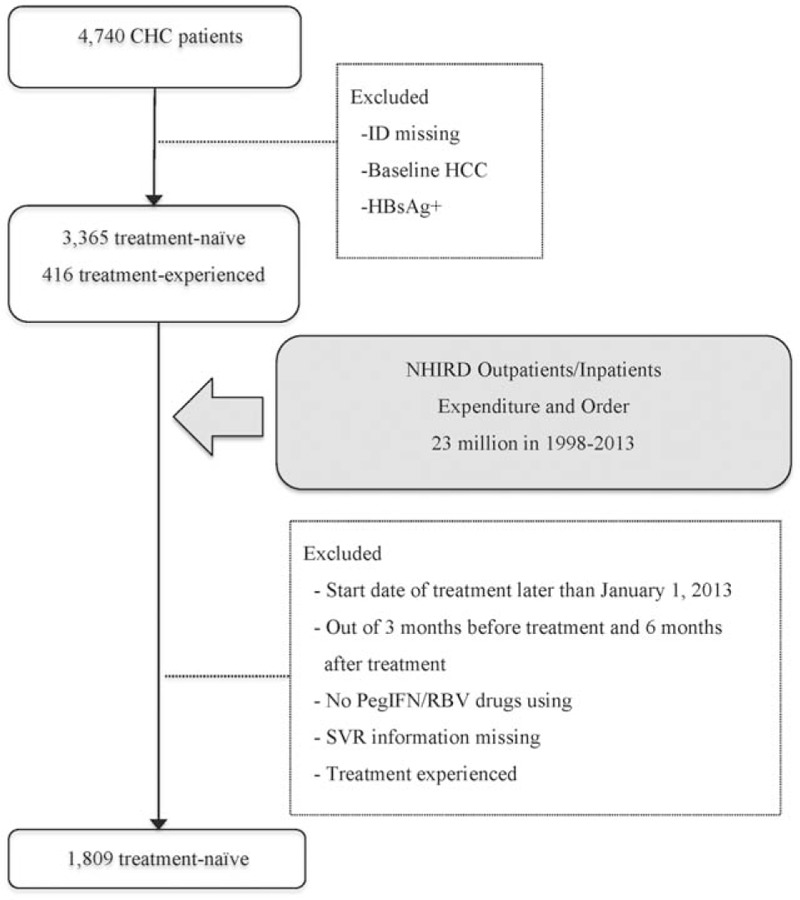
Enrolled study cohort. CHC = chronic HCV, HBsAg+ = positive hepatitis B virus, HCC = hepatocellular carcinoma, ID = identity number, NHIRD = National Health Insurance Research Database, PegIFN = pegylated interferon, RBV = ribavirin.

In this large cohort study, a total of 4740 CHC patients who ever had received PegIFN/RBV regimens were consecutively involved from a medical center and 2 core regional hospitals. All available on-treatment clinical data were assessed on the treatment courses. Patients with hepatocellular carcinoma before antiviral treatment, seropositive hepatitis B surface antigen, serum ANA titer >1:320, or with overt clinical manifestations or medical history related to autoimmune diseases were excluded. Therefore, 3781 CHC patients were further linked to the whole population of outpatient/inpatient expenditures and their orders of the NHIRD. The patients with meeting one of the following criteria were further excluded: the date of outpatient/inpatient visits were not within the assessed HCV management period; the start date of treatment was later than January 1, 2013; the patients were not using PegIFN/RBV therapies at each visit; SVR information was lack; or the patients were treatment-experienced. Finally, 1809 treatment-naive CHC patients were enrolled in the further cost-effective analysis (Fig. 1).

The protocols, followed the guidelines of the International Conference on Harmonization for Good Clinical Practice costs, were approved by the institutional review boards at the participating hospitals. All patients provided written informed consent.

### 2.2 Clinical data (laboratory tests and SVR assessment)

We used a qualitative real-time polymerase chain reaction (Cobas Amplicor Hepatitis C Virus Test 2.0, Roche, Branchburg, NJ)^[27]^ and a quantification branched DNA assay (Versant HCV RNA 3.0, Bayer, Tarrytown, NJ) with a detection limit of 615 IU/mL or real-time HCV (Abbott Molecular, Des Plaines, IL) with a detection limit of 12 IU/mL^[28]^ to detect serum HCV RNA. The HCV genotypes were determined using the Okamoto method^[29]^ or a real-time PCR assay (Abbott Real-Time HCV Genotype II; Abbott Molecular). Patients with mixed genotypes infection including G1 were classified as G1; those mixed with G2 but without G1 were classified as G2; the others were not included in the current study due to limited case numbers. The liver histology obtained within 1 year before antiviral therapy was classified and staged according to the scoring system described by Scheuer.^[30]^ A negativity of HCV RNA on a 24-week after treatment follow-up period was defined as achieved successful therapy, SVR. The IL28B rs8099917 genotype was determined by the method described in the previous study.^[31]^

### 2.3 Cost measurement from NHIRD

The inpatient- and outpatient-costs were calculated using NHIRD of the entire population from 1998 to 2013. The costs of prescribed medications, laboratory tests, consultations, logistics, and interventions retrieved from the linked NHIRD were calculated, respectively. The assessed period for the medical-care costs was retrieved between 3 months before the start of antiviral treatment and 6 months after the stop of antiviral treatment. The exchange rate of all medical-costs was converted by 32 New Taiwan dollars per US dollar.

### 2.4 Statistical analyses

The number and related percentage were presented in the calculation of category variables. The chi-square or Fisher exact tests were applied to test the difference between prevalence rates of the groups. Mean and its standard deviation (SD) were presented in the calculation of continuous variables. The student *t* test or ANOVA tests were applied to check the difference between the mean of more than 2 groups. We used the total number as a denominator to calculate the average inpatient cost (the proportion of patients who used inpatient services at least once in a given year). The average total cost per SVR achieved was calculated as the summation of the total cost for treated patients divided by SVR rate. The subgroup analysis of the average cost per SVR was stratified by HCV genotype. The specific subgroups were classified according to age (<40, 40–60, and ≥ 60), gender, baseline viral load (LVL; low viral load: ≤400 KIU/mL or HVL; high viral load: >400 KIU/mL), fibrosis staging (mild fibrosis: F0–1, moderate fibrosis: F2 or advanced fibrosis: F3–4), IL28B (TT and non-TT), rapid virologic response at week 4 (RVR; HCV RNA undetectable at week 4), and complete early virologic response at week 12 (cEVR; HCV RNA undetectable at week 12). All analyses were done using SAS software (SAS Institute Inc., Cary, NC) and according to 2-sided hypothesis tests with a significance level of *P* < .05.

## 3 Results

### 3.1 Demographic profile of all treatment-naïve CHC patients

**Figure 2:**
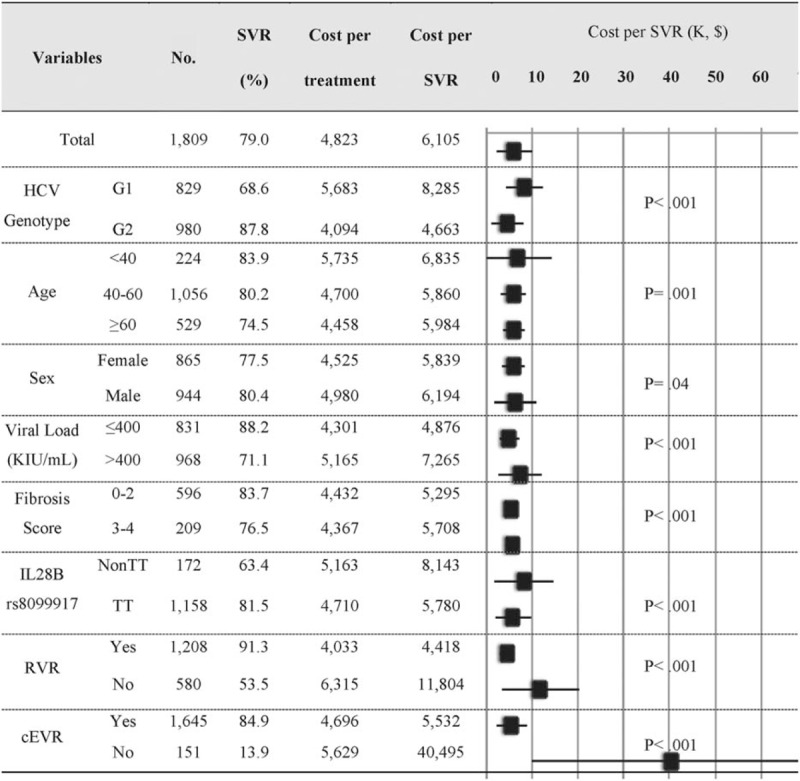
The costs of antiviral and nonantiviral agents, laboratory testing and consultation (US$), and the effectiveness evaluated as a sustained virological response (SVR) on entire treatment-naïve patients. Data were available in 1799 for viral load; 805 for histopathology; 1330 for IL28B rs8099917; 1788 for RVR; and 1796 for cEVR.

Among the 1809 CHC treatment-naive patients with a mean treatment duration of 28.3 weeks, 45.8% were infected with HCV G1, 70.8% were <60 years, 52.2% were males, and 74.1% had mild-moderate fibrosis (F0–2). The rate of RVR, cEVR, and SVR was 67.6%, 91.6%, and 79.0%, respectively (Fig. 2).

**Figure 3:**
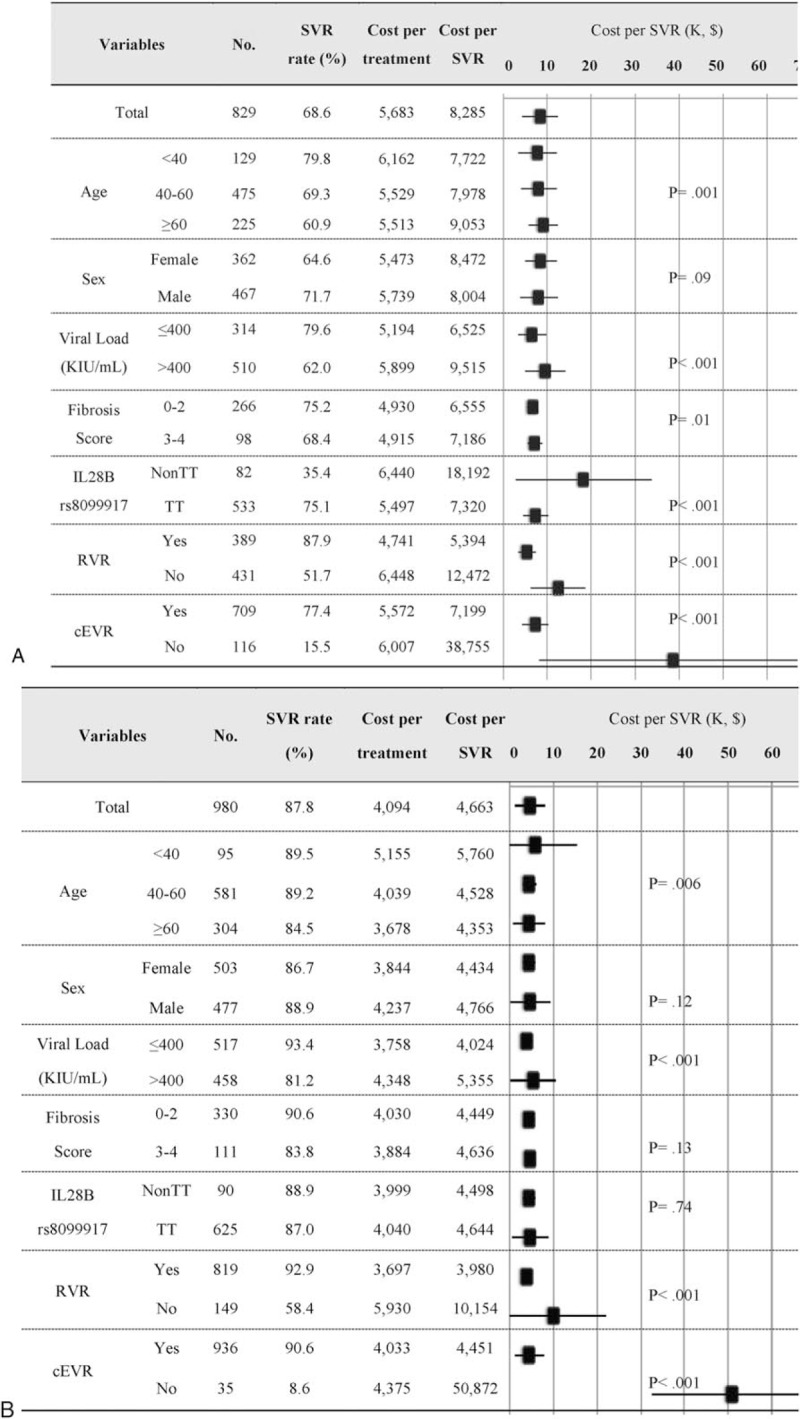
The cost of antiviral and nonantiviral agents, laboratory testing and consultation (US$), and the effectiveness evaluated as a sustained virological response (SVR) stratified by HCV genotype. (A) HCV G1 (n = 829), data were available in 824 for viral load, 364 for histopathology, 615 for IL28B rs8099917, 820 for RVR, and 825 for cEVR. (B) HCV G2 (n = 980), data were available in 975 for viral load, 441 for histopathology, 715 for IL28B rs8099917, 968 for RVR, and 971 for cEVR.

The HCV G1 patients had a significantly higher proportion of high baseline viral load (>400 KIU/mL) (61.9% vs 47.0%, *P* < .001) and longer treatment duration (mean: 35.3 vs 22.4 weeks, *P* < .001, Table 1) than the HCV G2 patients. The rates of RVR, cEVR, and SVR were 47.4%, 85.9%, and 68.6%, respectively, in HCV G1 patients, which were all significantly lower than those in HCV G2 patients (84.6%, 96.4%, and 87.8%, respectively, all *P* < .001) (Fig. 3A and B).

**Table 1:**
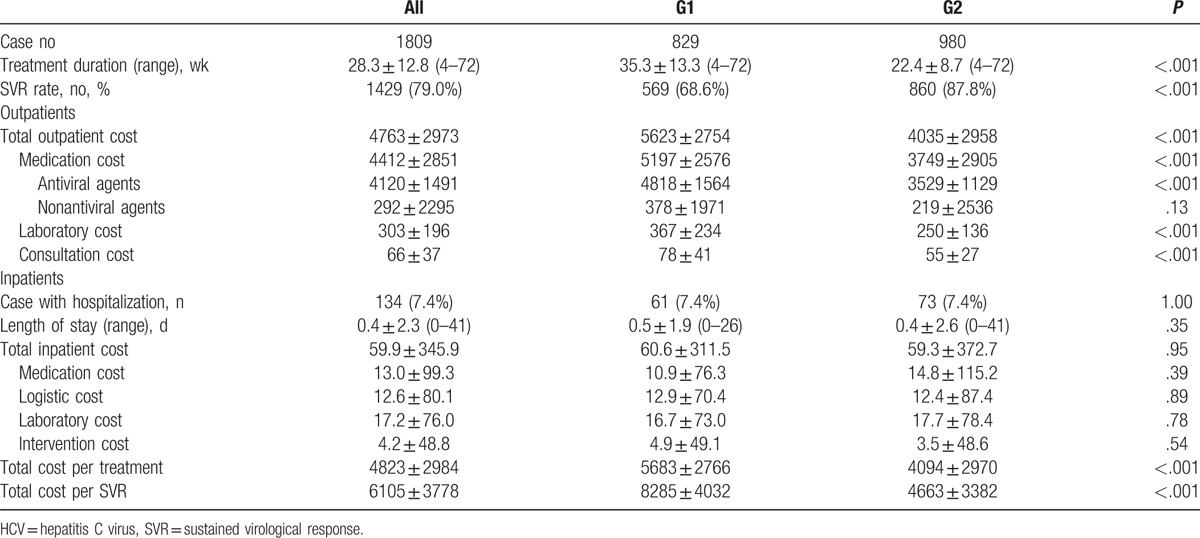
Outpatient- and inpatient-costs per treatment on treatment naïve, HCV genotype 1 (G1) and genotype 2 (G2) patients, respectively.

### 3.2 Medical-care cost of per treatment and per SVR for all treatment-naïve CHC patients

The average medical-care cost per treatment was $4823 (SD, 2984). A total of $4120 for antivirals agents, $292 for nonantivirals medication, $303 for laboratory testing, and $66 for consultation were included on the outpatient costs. There was an average inpatient cost of $60 of all 134 (7.4%) patients had hospitalizations (Table 1). The average itemized cost per SVR was $7023 for antivirals, $551 for nonantivirals, $535 for laboratory testing, and $114 for consultations in HCV G1 patients, which were significantly higher than those in HCV G2 patients ($4019, $249, $285, and $63, respectively, *P* < .001 for antivirals, .026 for nonantivirals, <.001 for laboratory testing, and <.001 for consultations). However, the inpatient costs were similar between HCV G1 and G2 patients ($88 and $67, respectively, *P* < .29).

The average total cost per treatment was $4823 for the entire population, which was significantly higher for HCV G1 patients than for HCV G2 patients ($5683 vs $4094, respectively, *P* < .001). The cost per SVR achieved was $6105 (SD, 3778) for all HCV treatment-naïve patients with an SVR rate of 79.0%. There was significantly higher in HCV G1 than in HCV G2 patients ($8285 and $4663, respectively, *P* < .001).

### 3.3 Subgroups analysis of cost-effectiveness for all treatment-naïve HCV patients

In addition to significantly higher cost-effectiveness of treatment in HCV G1 patients, patients who were young and male, with a high viral load, advanced fibrosis (F3–4), IL28B rs8099917 non-TT, no RVR achieved, or no cEVR achieved were the least cost-effectiveness patients to treat. The average cost per SVR achieved was $6835 for <40 years and 5901 for ≥40 years, respectively (*P* < .001); $6194 and $5839 for men and women, respectively (*P* = .043); $7265 and $4876 for higher and lower viral loads, respectively (*P* < .001); $5708 and $5295 for F3–4 and F0–2, respectively (*P* < .001); $8143 and $5780 for IL28B rs8099917 non-TT and TT, respectively (*P* < .001); $11,804 and $4418 for non-RVR and RVR, respectively (*P* < .001); and $40,495 and $5532 for non-cEVR and cEVR, respectively (*P* < .001) (Fig. 2).

### 3.4 Subgroup analysis of cost-effectiveness, stratified by the HCV genotype

The HCV genotype is the most important predictor of HCV treatment efficacy of PegIFN/RBV. Therefore, we further analyzed the cost-effectiveness of therapy stratified by the viral genotype **(**Fig. 3A and B**)**.

The subgroups of patients with favorable viral characteristics, including LVL, RVR, and cEVR had significantly lower average costs per SVR achieved when compared with their counterparts due to much lower SVR rates no matter in HCV G1 or HCV G2 groups. Patients without cEVR had the highest cost per SVR achieved, followed by those without RVR and those with high viral loads in both of HCV G1 and G2 ($38,755, $12,472, and $9515, respectively, in HCV G1 and $50,872, $10,154, and $5355, respectively, in HCV G2).

Regarding the impact of hepatic fibrosis, in the G1 group, higher treatment costs per SVR were observed in the patients with higher scores, when compared with patients with lower scores (*P* = .91 for the cost per treatment and 0.001 for the cost per SVR, Fig. 3A). However, the trend of a lower cost per SVR in the less fibrotic group was not observed in the G2 group (*P* = .16 for the cost per treatment and 0.13 for the cost per SVR, Fig. 3B).

HCV G1 patients carrying with favorable IL28B rs8099917 had a much higher SVR rate than those without (35.4% for non-TT and 75.1% for TT), but the difference did not exist in HCV G2 patients (88.9% for non-TT and 87.0% for TT). The role of host IL28B genotype on cost-effectiveness was only observed in HCV G1 patients. Patients with carrying unfavorable IL28B rs8099917 genotype had a significantly higher cost per SVR achieved, when compared with those who with carrying favorable IL28B rs8099917 ($18,192 and $7320, respectively, *P* < .001, Fig. 3A) in HCV G1 patients but not in HCV G2 patients ($4498 and $4644, respectively, *P* = .74, Fig. 3B).

Interestingly, age factors had different effects on cost-effectiveness between HCV G1 and G2 patients. In G1 patients, the SVR rate declined with older age (from 79.8% for age <40 years to 60.9% for age ≥60 years), but the cost per treatment was similar among the 3 age groups. Therefore, the cost per SVR achieved significantly increased with age, from $7722 among G1 patients of <40 years to $9053 among those of ≥60 years (*P* < .001, Fig. 3A). However, for G2 patients, the SVR rate was similar among age group. The cost per SVR achieved declined from $5760 for age <40 years to $4353 for age ≥60 years (*P* < .001, Fig. 3B) due to the cost per treatment decreased with age (from $5155 for age <40 years to $3678 for age ≥60 years).

## 4 Discussions

The current study was the first survey of the cost-effectiveness of HCV treatment in a large, real-world cohort, and total real-world expenses, including not only the direct but also the indirect costs of the outpatient and inpatients. We demonstrated that the average cost per SVR achieved for treatment-naïve CHC patients was $6105 in Taiwan. Patients with all of the traditionally unfavorable factors of SVR with except old age incurred much higher costs per SVR achieved. The cost per SVR was $8285 in treatment-naïve G1 CHC patients, which was 1.8 times the cost of $4663 in treatment-naïve G2 CHC patients. Treatment-naïve G1 patients of age ≥60 years, with viral loads >400 KIU/mL, carrying the IL28B unfavorable genotype, no achieving an RVR at week 4 or a cEVR at week 12 had much poorer cost effectiveness to PegIFN/RBV therapy, ranging from around $9000 to as high as $38,755 per SVR achieved. For treatment-naïve G2 patients, although baseline viral loads >400 KIU/mL and age <40 years had significantly higher costs per SVR achieved compared to their counterparts, the cost remained around $5500 per SVR. In comparison, for treatment-naive G2 patients without an RVR or cEVR, the cost per SVR achieved was 2 to 10 times the average cost of G2 patients, $10,154 and $50,872, respectively.

The current study demonstrated that the SVR rate in real-world clinical practice was 68.6% for HCV G1 and 87.8% for HCV G2 patients, which were substantially lower than the rates reported in our previous clinical trial.^[14,15]^ This difference might be due to the relatively strict patient selection criteria utilized in clinical trials. Also, the Taiwan Health Insurance Agency reimbursed for a 24-week regimen before 2009 regardless of viral genotype, which also led to the inferior SVR rate among HCV G1 patients with high baseline viral loads or who did not achieve an RVR.^[14]^ Similar to our previous report, the cost per SVR achieved of HCV G1 patients was about twice the cost of HCV G2 patients^[32]^ due to lower SVR rates and higher treatment costs despite longer treatment durations for HCV G1 patients.

In the current study, although female patients had lower SVR rates than male patients (77.5% vs 80.4%), the cost per SVR achieved was significantly lower in female than in male patients ($5839 vs $6194, respectively, *P* = .04). This is due to the lower cost per treatment of female patients due to lower body weights and therefore lower RBV dose exposures.

For HCV G1 patients, patients with traditionally unfavorable factors for SVR including old age, high baseline viral loads, advanced fibrosis, IL28B non-TT genotype, not achieving an RVR, and not achieving a cEVR incurred significantly higher costs per SVR achieved than their counterparts. The cost per SVR increased from $9000 to $38,755, indicating that these patient populations might consider the newly introduced DAA. Nevertheless, we recently demonstrated that patients over 40 years of age or with at least stage 2 fibrosis are at increased risk for HCC development overtime if left untreated.^[33]^ This patient population should be treated as early as possible even at a high cost per SVR achieved. These findings might echo the use of our previously proposed concept, resource-guided therapy, in prioritizing the HCV treatment in areas with limited resources.^[25]^

For HCV G2 patients, patients without an RVR, without a cEVR, and with high baseline viral loads had significantly higher costs per SVR achieved than observed in their counterparts. Nevertheless, although F3–4 G2 patients had lower SVR rates than F0–2 G2 patients, the cost per SVR achieved was not different between the 2 groups. This lack of difference occurs because the F3–4 G2 patients had lower cost pretreatments, which might be due to the higher adverse events, such as neutropenia, anemia, and thrombocytopenia,^[16]^ leading to dose adjustments among patients with advanced liver diseases. Because only some patients received liver biopsies for fibrosis status examination, the selection bias for liver biopsies, such as including healthier patients and those with lower bleeding risks, would be a contributing confounding factor in the cost per treatment per fibrotic group. Since IL28B had no impact on HCV G2 efficacy to PegIFN/RBV,^[13]^ the cost per SVR remained similar between the different IL28B genotypes. Interestingly, patients aged ≥60 years had a lower SVR rate but also a lower cost per treatment, resulting in a decreased cost per SVR achieved with an increase in age in HCV G2 patients. This finding might also be due to more dose adjustments in the older patients who had minor impacts on G2 efficacy to PegIFN/RBV.^[34]^ In G1 patients, a reduction in treatment duration or dosage will significantly ameliorate the SVR,^[14]^ therefore, although older patients had lower observed costs per treatment, the decline of SVR consequently resulted in a higher cost per SVR. However, in the G2 groups, dose reductions or shorter treatment duration was equally effective or only minor was less effective with respect to SVR,^[15]^ but the cost per treatment declined with age; hence, to our surprize, we found a significantly lower cost per SVR in G2 patients in contrast to the seemingly reasonable higher cost per SVR in older G1 patients. These results suggested that treatment of more elderly patients is cost-effective and justified with PegIFN/RBV.

Recent studies showed that 2 copies of the T allele (TT genotype) for the IL28B SNP rs8099917 were associated with higher SVR rates among HCV G1 patients, which contributed to lower medical costs.^[35,36]^ Our previous study demonstrated that HCV G1 patients carried with the favorable IL28B genotype and with lower baseline viral loads had high positive predictive values for SVR from a 24-week regimen of PegIFN/RBV.^[12]^ The findings of the present cost-effective analysis may support our previous results.

Therefore, in a real-world cost effective analysis, we highlighted subgroups of extremely high cost per SVR achieved (defined as >mean + SD), including HCV G1 patients carrying with the unfavorable IL28B genotypes, those without RVR, and those without cEVR; HCV G2 patients without RVR or cEVR. However, the current study evaluated the cost-effectiveness of PegIFN/RBV treatment only based on the direct medical and nonmedical costs and indirect medical costs. Further studies including measurement of indirect and intangible costs, such as psychologic stress, work productivity reduction due to absenteeism and presenteeism, as well as the other assessments of quality of life are warranted to have a comprehensive comparison between IFN-based and IFN-free regimens for CHC treatment.

In conclusion, the current study revealed the real-world cost effectiveness of PegIFN/RBV for treatment-naïve CHC patients. PegIFN/RBV in the treatment-naïve patients without cEVR, who have not achieved RVR, or those with HCV G1 with unfavorable IL28B genotypes were cost effective. Our results could provide evidence for policy-makers deciding on the strategies for the use of costly DAA for treating HCV patients in the near future.
